# *Euterpe oleracea* extract (açaí) exhibits cardioprotective effects after chemotherapy treatment in a breast cancer model

**DOI:** 10.1186/s12906-023-04104-7

**Published:** 2023-08-25

**Authors:** Jéssica Alessandra-Perini, Daniel Escorsim Machado, Celia Yelimar Palmero, Marllow Caetano Claudino, Roberto Soares de Moura, Antônio Palumbo Junior, Jamila Alessandra Perini, Luiz Eurico Nasciutti

**Affiliations:** 1 Instituto de Ciências Biomédicas (ICB), Universidade Federal do Rio de Janeiro (UFRJ), Rio de Janeiro, RJ Brazil; 2 Laboratório de Pesquisa em Ciências Farmacêuticas (LAPESF), Universidade do Estado do Rio de Janeiro (UERJ), Manuel Caldeira de Alvarenga Avenue, 1.203, Rio de Janeiro, RJ 23070-200 Brazil; 3 Laboratório Integrado de Morfologia, Instituto de Biodiversidade e Sustentabilidade, Universidade Federal do Rio de Janeiro 9UFRJ), Rio de Janeiro, RJ Brazil; 4 Departamento de Farmacologia e Psicobiologia, Universidade do Estado do Rio de Janeiro (UERJ), Rio de Janeiro, RJ Brazil

**Keywords:** Breast cancer, *Euterpe oleracea*, Açaí, Cardioprotection, Doxorubicin

## Abstract

**Background:**

Açaí, a Brazilian native fruit, has already been demonstrated to play a role in the progress of breast cancer and cardiotoxicity promoted by chemotherapy agents. Thus, the present study aimed to evaluate the combined use of açaí and the FAC-D chemotherapy protocol in a breast cancer model in vivo.

**Methods:**

Mammary carcinogenesis was induced in thirty female Wistar rats by subcutaneous injection of 25 mg/kg 7,12-dimethylbenzanthracene (DMBA) in the mammary gland. After sixty days, the rats were randomized into two groups: treated with 200 mg/kg of either açaí extract or vehicle, via gastric tube for 45 consecutive days. The FAC-D protocol was initiated after 90 days of induction by intraperitoneal injection for 3 cycles with a 7-day break each. After treatment, blood was collected for haematological and biochemical analyses, and tumours were collected for macroscopic and histological analyses. In the same way, heart, liver, and kidney samples were also collected for macroscopic and histological analyses.

**Results:**

Breast cancer was found as a cystic mass with a fibrotic pattern in the mammary gland. The histological analysis showed an invasive carcinoma area in both groups; however, in the saline group, there was a higher presence of inflammatory clusters. No difference was observed regarding body weight, glycaemia, aspartate aminotransferase (AST), alanine aminotransferase (ALT), creatinine, and urea in either group. However, açaí treatment decreased creatine kinase (CK), creatine kinase MB (CKMB), troponin I and C-reactive protein levels and increased the number of neutrophils and monocytes. Heart histopathology showed normal myocardium in the açaí treatment, while the saline group presented higher toxicity effects with loss of architecture of cardiac tissue. Furthermore, the açaí treatment presented greater collagen distribution, increased hydroxyproline concentration and lower H2AX immunostaining in the heart samples.

**Conclusion:**

Açaí decreased the number of inflammatory cells in the tumor environment and exhibited protection against chemotherapy drug cardiotoxicity with an increased immune response in animals. Thus, açaí can be considered a promising low-cost therapeutic treatment that can be used in association with chemotherapy agents to avoid heart damage.

## Introduction

In 2020, female breast cancer has surpassed lung cancer as the most commonly diagnosed cancer worldwide, with approximately 2.3 million new cases, accounting for 15.4% of all new cancer cases [1]. In women, breast cancer is the most diagnosed cancer, excluding nonmelanoma skin tumours, corresponding to 24.5% of cases worldwide [1]. Moreover, it represents the leading cause of cancer death in women, corresponding to 25% worldwide [2]. In Brazil, there was 15.403 deaths for breast cancer in 2015 [3], and 66.280 new cases of breast tumor in women are expected for each year in the 2020–2022 biennium [4], which makes breast cancer an important health issue in Brazil.

The main strategies used in breast cancer therapy are surgery, radiotherapy, chemotherapy, hormone therapy, and targeted therapy [5, 6]. The mainstream current protocol used in Brazil, called “FAC-D”, is based on 5-fluorouracil, doxorubicin, and cyclophosphamide [7–10], sometimes combined with other agents such as docetaxel or paclitaxel [9]. Importantly, these protocols produce many side effects, which negatively impact the woman’s quality of life and the success of the treatment [6, 11]. One of the main and most well-documented adverse effects of anthracyclines, such as doxorubicin, is cardiac toxicity [12–14], which limits its clinical utility [15–17]. Patients who received doxorubicin treatment had an estimated incidence of cardiac events of 10–25% and a prevalence of left ventricular contractile dysfunction of 50–60% using a cumulative dose of doxorubicin (430–600 mg/m^2^) [18, 19]. Thus, new therapies to reduce the adverse effects of current treatments have become of increasing interest for the management of women with breast cancer.

Açaí (*Euterpe oleracea* Mart.) is an economically important palm fruit that is native to the Brazilian Amazon [20]. This fruit has received significant attention in recent years due to its pharmacological properties such as anti-inflammatory, antioxidant, cardioprotective and anticancer activities [21–28]. Recently, our group demonstrated an antiangiogenic, anti-inflammatory and anticarcinogenic effect of açaí in a breast cancer rat model [21]. Moreover, in malignant breast cancer cells, açaí demonstrated an antitumorigenic effect by increasing the autophagy process, decreasing cellular viability [27, 29, 30] and necroptosis [29] and increasing the reactive oxygen species production pathway [30]. In addition, açaí also exhibited a cardioprotective effect after treatment with chemotherapeutic doxorubicin [31, 32], which promotes severe cardiotoxicity [33].

According to the evidence that açaí may modulate the progression of breast cancer and reduce the cardiotoxicity promoted by chemotherapy agents, we evaluated the combined use of this extract and the current FAC-D chemotherapy protocol in a breast cancer experimental model.

## Methods

### Preparation of the extract from açaí

*Euterpe oleracea* Mart. fruits were obtained from the Amazon Bay (Belém do Pará, Pará, Brazil; excicata number MG 205.222, Museu Paraense Emílio Goeldi, Belém do Pará, Pará, Brazil). The hydroalcoholic extract was obtained from decoction of the açaí stones, as previously described [21, 23, 26, 27, 34]. Briefly, açaí stones (200 g) were boiled in distilled water for 10 min and the decoction was allowed to cool at room temperature and then extracted by shaking with ethanol (400 mL) and kept at 4 °C for 10 days. Then the extract was filtered (using a Whatman filter paper) and the ethanol was evaporated (Fisatom Equipamentos Científicos Ltda São Paulo, São Paulo, Brazil) under low pressure at 55 °C. The extract was lyophilized (Fisatom Equipamentos Científicos Ltda São Paulo), and frozen at -20 °C until use. The composition of açaí used was performed previously analysis indicating the presence of catechin and polymeric pro-anthocyanidins [25].

### Breast cancer experimental model

All experiments were carried out in accordance with the Ethical Guidelines from the Institutional Animal Care and Use Committee (CEUA) and the NIH Guidelines for the Care and Use of Laboratory Animals (http://oacu.od.nih.gov/regs/index.htm. 8th Edition; 2011). The protocol used was approved by the CEUA of the West Zone State University (UEZO) with protocol code 008/2019. The animals were housed in polyethylene cages at the animal facility and were maintained under a 12-h light/dark cycle with a controlled temperature (25 °C) and free access to water and food.

Thirty female Wistar rats (150–200 g and 8-week-old) were used for the experimental induction of breast cancer using the method described by Deepalakshmi and Mirunalini [35] and our group [21, 36]. In summary, the rats were anesthetized (ketamine and xylazine) and the breast tumour was induced by subcutaneous injection in the mammary region with one single dose of 25 mg/kg 7,12-dimethylbenzanthracene (DMBA) (Sigma-Aldrich, St. Louis, MO) dissolved in 0.5 mL of physiological saline and 0.5 mL of sunflower oil. The DMBA was used according to the manufacturer’s instructions. Rats were palpated in the mammary gland once a week to detect the presence of breast tumours for 15 weeks.

### Açaí and cytotoxic chemotherapy treatment

**Fig. 1:**
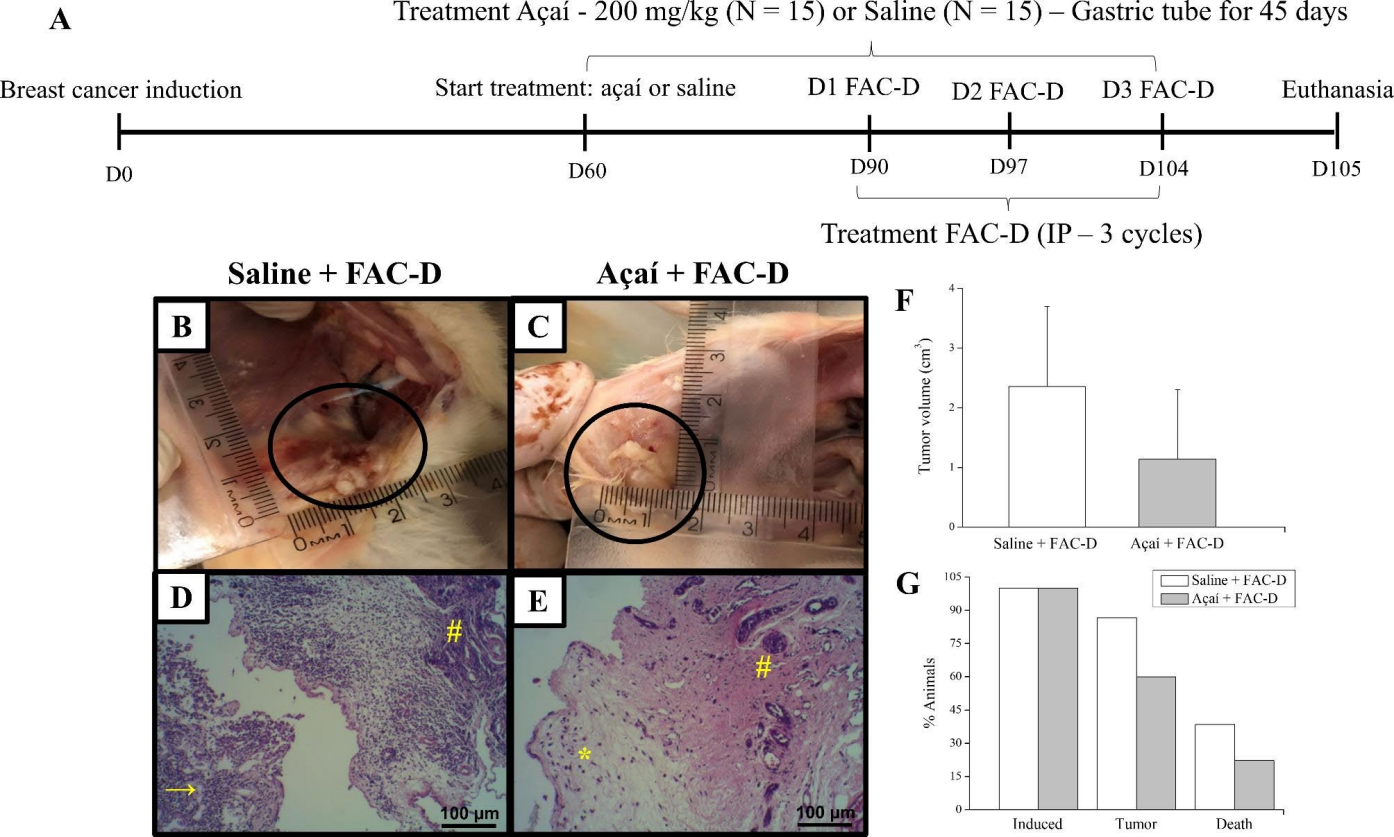
Morphological characteristics of breast cancer in the saline + FAC-D and açaí + FAC-D treated groups. Timeline representation of the experimental model (**A**). Macroscopic view of breast cancer after treatments (**B, C**). DMBA induced breast cancer in the saline + FAC-D (**B, D**) and açaí + FAC-D (**C, E**) groups. There was no significant difference in tumour volume (**F**), P-value 0.085. Histological analysis (**D, E**) showed the presence of carcinoma invasion areas in both groups, with hyperchromatic nuclei cells consistent with a mononuclear inflammatory infiltrate in the fibrovascular stroma (#). However, a higher number of inflammatory cells (→) in the saline + FAC-D group (**D**) and a decreased number of these cells (*) in the açaí + FAC-D group (**E**) were observed. Tumour incidence (P-value 0.09) and mortality (P-value 0.42) in the saline + FAC-D and açaí + FAC-D treatment groups are shown (**G**)

Sixty days after tumour induction, the animals were randomly divided into two groups of 15 rats each: the açaí group was treated with 200 mg of açaí per kg of body weight dissolved in saline, and the control group received saline only. The dose was based on previous studies [21, 23, 37, 38], including significant results using 200 mg/kg açaí, which prevented the carcinogenesis induced by DMBA and increased the overall survival of rats with breast cancer [21]. Both treatments were administered daily by gastric tube for 45 consecutive days. Thirty days after beginning the açaí or saline treatment, the animals received intraperitoneal injection of the FAC-D protocol (75 mg/kg of 5-fluorouracil + 15 mg/kg of doxorubicin + 10 mg/kg of cyclophosphamide + 10 mg/kg of docetaxel). This preparation was administered in three doses at 7-day intervals each. Body weight and glycaemia were measured before the açaí treatment and every seven days until the last day of treatment, when the animals were euthanized by anaesthesia overdose (ketamine and xylazine). The experimental model representation is in Fig. 1A. The blood samples were collected for biochemical and hematological analyses. All visible mammary tumours were counted, excised, weighed, and measured (length x width). The tumour volumes were calculated according to the following formula: Tumour volume = 1/2 (length x width^2^). In addition, the heart, liver, and kidneys were collected, weighed, measured (length x width) and fixed in 10% buffered formalin and embedded in paraffin for histological and immunohistochemical studies.

### Histology, immunohistochemistry, and morphometric analyses

Formalin-fixed tumour and cardiac tissues were paraffin-embedded and cut into 4-micrometers-thick sections for histology. Furthermore, cardiac tissues were used for immunohistochemical analyses. Part of the sections were stained with Harris hematoxylin and eosin (HE), and examined under light microscope (Nikon, Tokyo, Japan) at 200x. For observation of collagen fibres distribution, additional cardiac sections were stained with picrosirius red and the percentage of the marked area in reddish-yellow by field was calculated with the Image Pro Plus 4.5.1 (Media Cybernetics, Silver Spring, MD). The other paraffin-embedded cardiac tissues sections were placed on silane-treated slides and incubated with polyclonal antibody against gamma H2A.X (ab-2893, Abcam, Cambridge, UK) at 1:400 dilution overnight. Negative control slides consisted of sections incubated with antibody vehicle. Then, the sections were revealed using LSAB2 Kit (HRP, rat, Dako-Cytomation, Carpinteria, CA, USA) with diaminobenzidine (3,3’-diaminobenzidine tablets; Sigma, St. Louis, MO) as the chromogen and counterstained with hematoxylin. All slides were examined by two blinded observers using a 40× objective lens on a light microscope (Nikon, Tokyo, Japan) connected to a digital camera (Coolpix 990; Nikon) and ten fields of an immunostained section were chosen at random and captured with high-quality images (2048 × 1536 pixels buffer) and quantified using Image Pro Plus 4.5.1 (Media Cybernetics, Silver spring, MD). The histologic scores (H) for H2A.X were calculated as previously described [21, 23], using the formula H = ΣPi, where I is the intensity ranging from 0 (negative cells) to 3 (deeply staining cells) and P is the percentage of staining cells for each given i, with P values of 1, 2, 3, 4, and 5 indicating < 15%, 15–50%, 50–85%, > 85%, and 100% positive-staining cells. The staining result was expressed as mean ± standard deviations.

### Hematological and biochemical analysis

The leukogram was performed using blood smears, stained (Panotico Fast, Laborclin, Brazil) and viewed under an optic microscope (Nikon, Japan) for differential count of neutrophils, lymphocytes, monocytes, eosinophils, and basophils. Aspartate aminotransferase (AST), alanine aminotransferase (ALT), creatinine, urea, creatine kinase (CK) creatine kinase MB (CKMB), and C-reactive protein were evaluated using the respective kits (K048-6, K049-6, K067-1, K056-1, K010, K069 and K059, Bioclin, MG, Brazil, respectively) and cardiac troponin I was determined using kit (Elabscience Biotechnology Co. Ltd., Wuhan, China). All biochemical concentrations were determined according to the instructions for each kit used. The concentration of hydroxyproline in the rat cardiac tissues were measured with an ELISA kit (ab222941 Abcam, Cambridge, UK) following the manufacturer’s protocol.

### Statistical analysis

Continuous data were expressed as means ± standard deviations and statistical analyses were performed using Student’s t test, while categorical data were expressed as percentages and evaluated by the Person Chi-square test. For H2AX morphometric analysis, statistical calculations were performed using the Stat-Xact-5 software program (CYTEL Software Corporation, Cambridge, MA, USA). Differences with P values ≤ 0.05 were considered statistically significant.

## Results

### Açaí is effective in reducing breast tumour growth

After 45 days of treatment, both groups had mammary tumour growth in a cystic pattern and adhesions in a markedly fibrotic pattern (Fig. 1B and C); however, no significant differences in tumour weight (saline + FAC-D (n = 8): 1.07 ± 0.91 g; açaí + FAC-D (n = 7): 0.45 ± 0.37 g, P-value 0.117) and volume (saline + FAC-D (n = 8): 2.36 ± 1.34 g; açaí + FAC-D (n = 7): 1.14 ± 1.17 g, Fig. 1F) were noted. Histopathological analysis did not show significant differences in the area of invasive carcinoma in both groups; however, the presence of inflammatory clusters was higher in the saline + FAC-D group (Fig. 1D) than in the açaí + FAC-D group (Fig. 1E). The tumour incidence rates were approximately 87% and 60% in the saline + FAC-D and açaí + FAC-D groups, respectively. Throughout the experiment 5 animals from the saline + FAC-D group died (33% mortality rate) while in the açaí + FAC-D group, only 2 animals died (13% mortality rate) (Fig. 1G). Moreover, saline + FAC-D animals had a 1.44-fold higher risk of tumour development than açaí + FAC-D rats. Finally, the risk of dying was 1.73-fold higher among the group of animals that developed tumours.

### Açaí restores cardiac biochemical parameters and the number of neutrophils and monocytes

**Fig. 2:**
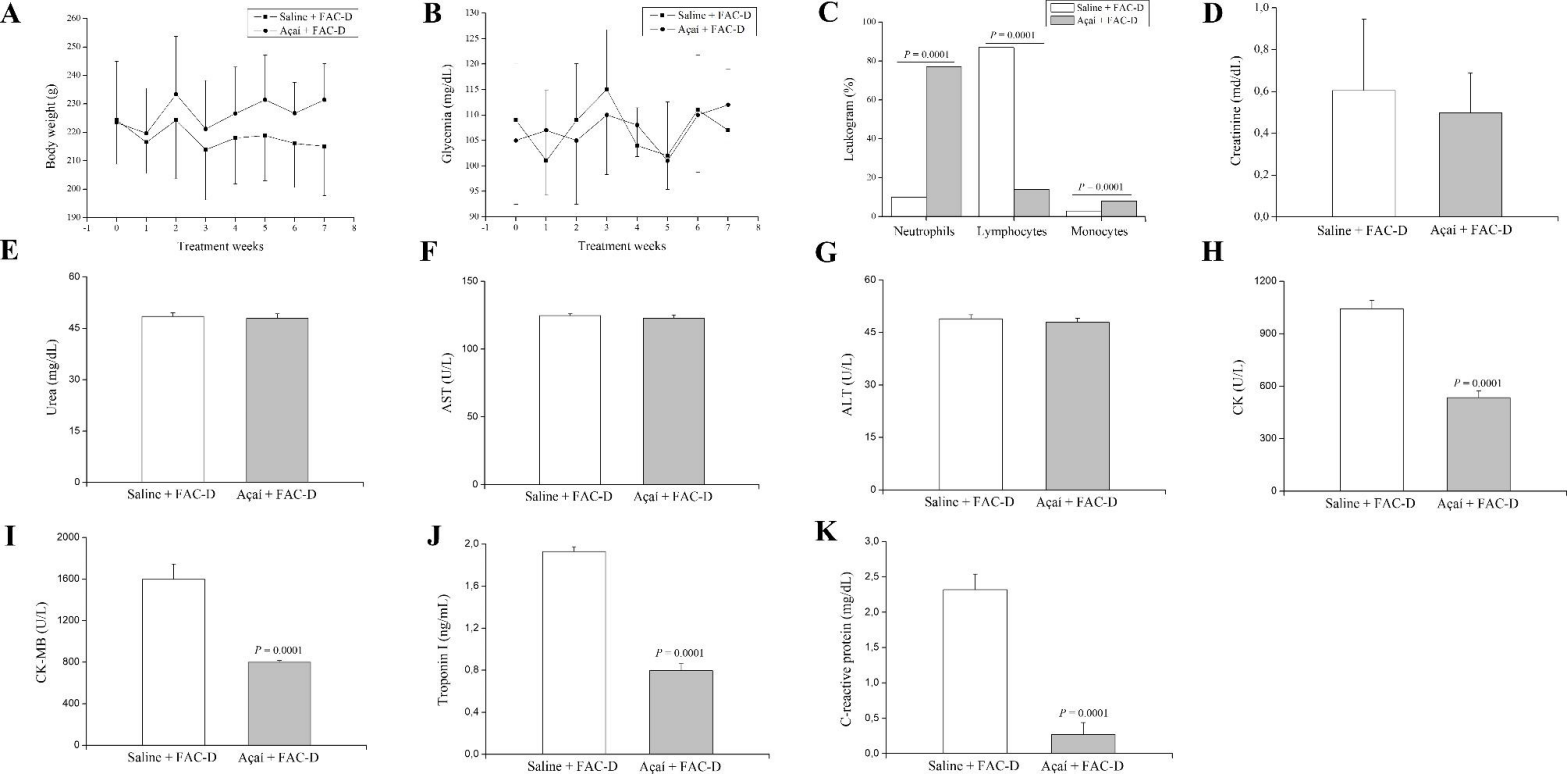
Biochemical parameters at the end of treatments with açaí or saline and the FAC-D protocol. No evidence of toxicity was noted between the açaí + FAC-D treated group and the saline + FAC-D group according to body weight (**A**), glycaemia (**B**) and, creatinine (**D**), urea (**E**), AST (**F**) and ALT (**G**) levels. In the haematologic analysis (**C**), lymphocytosis was observed in the saline + FAC-D animals, while in the açaí + FAC-D group, there was a recovery in the neutrophil number (**C**). CK (**H**), CKMB (**I**), troponin I (**J**), and C-reactive protein (**K**) levels were higher in saline + FAC-D animals than in açaí + FAC-D rats. Data are expressed as the mean ± standard deviation (saline + FAC-D: n = 8 and açaí + FAC-D: n = 7)

No differences in food consumption, body weight (Fig. 2A) and glycaemia (Fig. 2B) were observed in either group. However, in the haematologic analysis with peripheral blood, we observed an accentuated counting of lymphocytosis in saline + FAC-D animals, while in the açaí + FAC-D animals, the numbers of neutrophils and monocytes were increased (Fig. 2C). There were no significant differences in creatinine (Fig. 2D), urea (Fig. 2E), serum AST (Fig. 2F), and ALT (Fig. 2G) levels between the açaí + FAC-D and saline + FAC-D groups. Nevertheless, CK (Fig. 2H) and CKMB (Fig. 2I) serum levels decreased by approximately 50% in the açaí + FAC-D group compared to the saline + FAC-D group. Furthermore, troponin I (Fig. 2J) and C-reactive protein (Fig. 2K) serum levels were reduced by 58% and 88%, respectively, in the açaí + FAC-D group compared with the saline + FAC-D group.

### Açaí extract exerts a protective effect on the heart structure

**Fig. 3:**
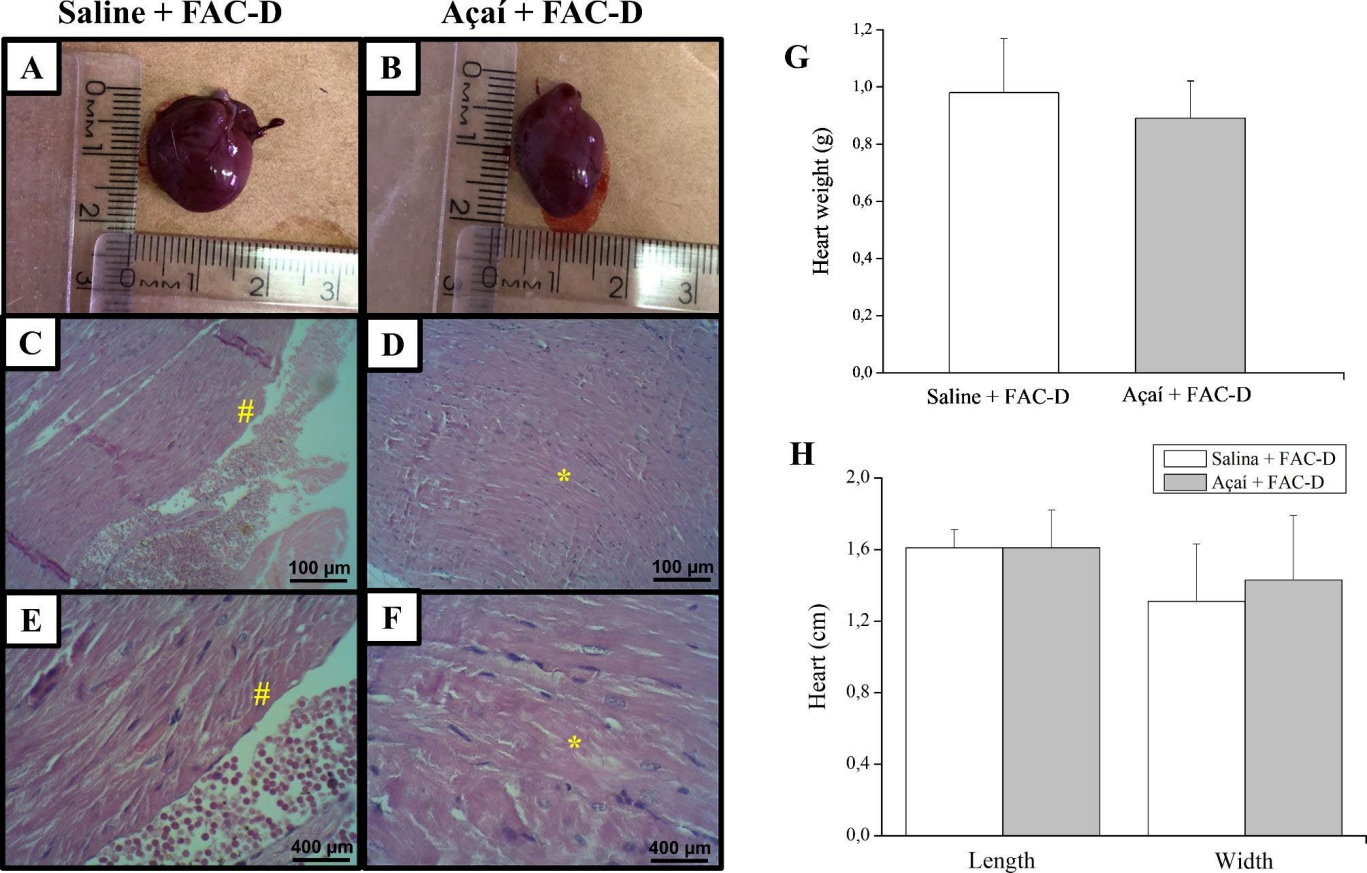
Açaí reduces chemotherapy-induced cardiotoxicity in experimental breast cancer. No macroscopic differences were observed in the saline + FAC-D (**A**) and açaí + FAC-D (**B**) groups, nor in the weight (**G**) and size (**H**) of the heart. Microscopic analysis showed increased toxicity with extensive damage in cardiac tissue architecture and muscle fibre loss (#) in the saline + FAC-D group (**C, E**); however, in the açaí + FAC-D group (**D, F**) only normal myocardial morphology was observed (*). Data are expressed as the mean ± standard deviation (saline + FAC-D: n = 8 and açaí + FAC-D: n = 7)

The macroscopic analysis of the heart (Fig. 3A and B) did not show significant differences regarding weight (Fig. 3G) and length or width (Fig. 3H) in both groups, while the histopathological analysis revealed higher toxicity effects with loss of cardiac tissue architecture in the saline + FAC-D group (Fig. 3C and E). However, açaí treatment markedly ameliorated the chemotherapy-induced pathological changes in the cardiac tissue and restored normal myocardial histology (Fig. 3D and F).

**Fig. 4:**
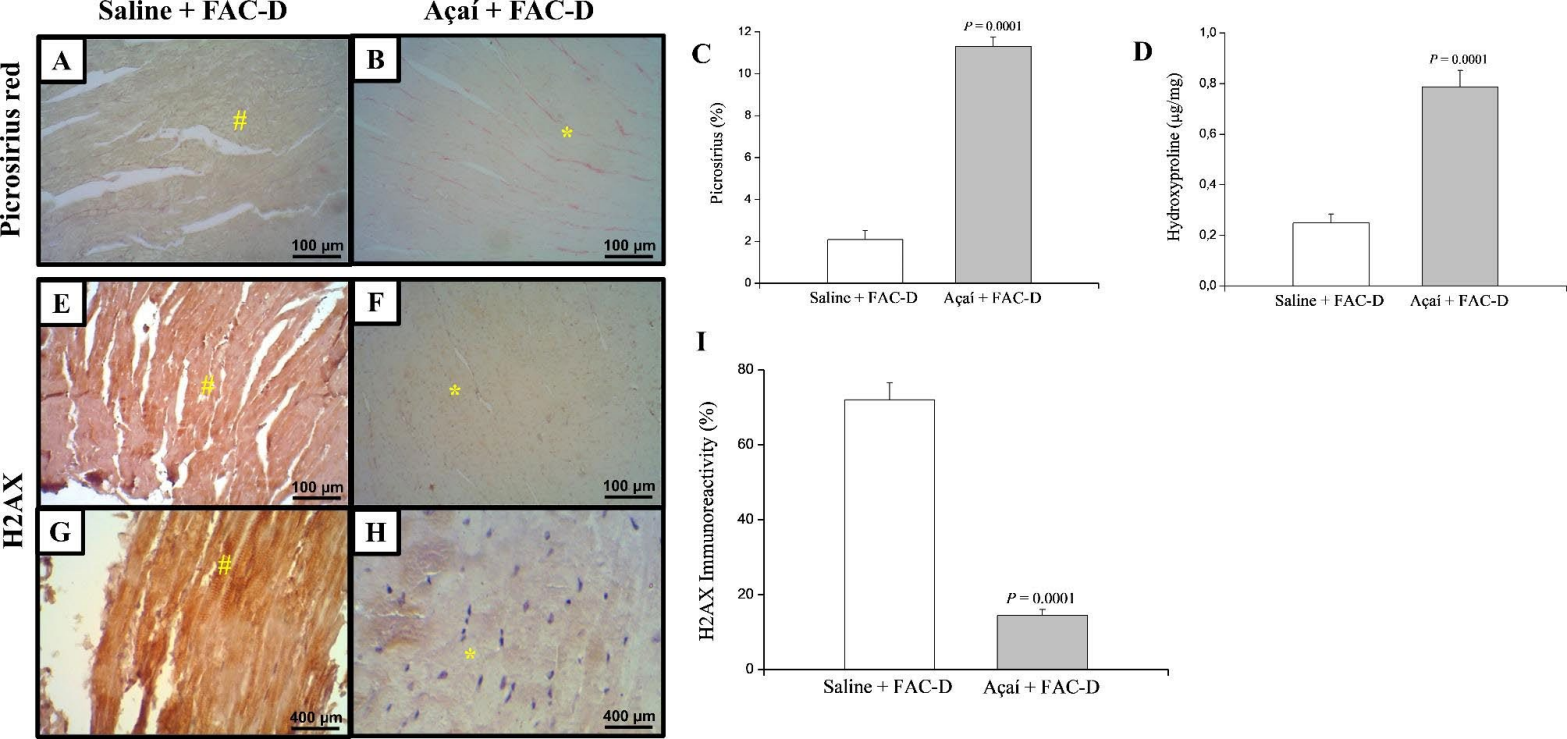
Açaí decreases DNA damage in heart cells. Picrosirius red staining was more intense and more evenly distributed in heart tissue samples from the açaí + FAC-D rats (**B**) than in those from the saline + FAC-D rats (**A**), and the difference in terms of collagen deposition was confirmed by histomorphometric analysis (**C**). The hydroxyproline concentration was higher in the açaí + FAC-D group than in the saline + FAC-D group (**D**). The immunoreactivity of H2AX was predominantly detected in the hearts of those in the saline + FAC-D group (**E, G**) compared with those of the açaí + FAC-D group (**F, H**). **I**. Histomorphometry evaluations of H2AX are shown

Picrosirius staining demonstrated a significant increase in collagen production in the açaí + FAC-D treatment group (Fig. 4B) compared with the saline + FAC-D group (Fig. 4A). These observations were confirmed by the percentage of area occupied by collagen fibres, evidencing that açaí increased this area by approximately 80% compared with the control group (Fig. 4C), and the hydroxyproline concentration was higher in the açaí + FAC-D group than in the saline + FAC-D group (Fig. 4D).

To investigate the cardioprotection in the açaí + FAC-D group, we performed H2AX immunohistochemistry analyses, which is a cellular damage marker in response to DNA (Deoxyribonucleic acid) double-strand breaks [39]. The immunohistochemical analysis revealed high H2AX staining in the heart samples from the saline + FAC-D group (Fig. 4E and G), while a weak staining pattern was observed in the heart samples from the açaí + FAC-D treated group (Fig. 4F and H). Moreover, the histomorphometry evaluations of DNA damage markers confirmed a significant decrease of approximately 80% in the açaí group compared with the saline group (Fig. 4I).

## Discussion

Throughout the history of civilization, natural products have played a prevailing role in the treatment of human diseases, including cancer [40]. Of the 1562 new drugs approved by the Food and Drug Administration between 1981 and 2014, 38.1% were natural or derived from natural products [41]. Therefore, naturally derived products represent an important resource for pharmaceutical companies that develop new medicines [42, 43]. With more than 50 thousand species of higher plants, Brazil has the largest biodiversity in the world; however, few pharmacological products have been developed from active components derived from Brazil [42]. Açaí is a fruit abundantly found in the Amazon region of Brazil [43] that has demonstrated antioxidant, anti-inflammatory, anticancer, cardioprotective and antinociceptive activities [21–28], and has been reported as a “natural” therapeutic option in the treatment of several diseases, such as breast cancer [21, 27, 29, 30].

Pretreatment with açaí could prevent the chemical carcinogenesis induced by the chemical agent DMBA, as previously shown that 112 days of açaí consumption significantly increased the overall and cumulative survival of rats with breast cancer [21]. Although in this study, the antitumorigenic effect of açaí was not observed, probably because açaí was administered 60 days after breast cancer induction with DMBA, our results indicate that açaí diminishes the inflammatory process in the breast cancer microenvironment, reducing the number of inflammatory cells. Tumorigenic pathways are not the only pathways involved in breast tumour development, suggesting a relevant participation of the tumour inflammatory microenvironment [44, 45]. Notably, 45 days of treatment with 200 mg/kg açaí did not affect the animal’s food consumption, body weight or glycaemia, as was previously described in colon carcinogenesis treated with a diet containing 2.5% or 5% açaí for 10 weeks or 5% açaí for 20 weeks [46] and in urinary bladder carcinogenesis treated with a standard diet with 2.5% and 5% açai for 10 weeks or a diet containing 5% açaí for three weeks [47]. Furthermore, no alterations in hepatic and kidney enzymes in the serum were observed, which is consistent with data from Cordeiro et al. [48] on diabetes induction and Da Costa et al. [49] syndrome in rats with renovascular hypertension. Thus, these results are strong indicators that açaí reduces the inflammatory process without harming the liver or kidney of the animals and support its utilization to prevent the side effects of the FAC-D protocol in breast cancer treatment.

Our group already showed the anti-inflammatory activity of açaí in breast cancer [21], in acute lung inflammation induced by cigarette smoke [26] and diabetic rats [50], accordingly as observed in individuals with metabolic syndrome [51] and other experimental models, including cancer [22, 52, 53]. In addition, açaí pulp supplementation doses of 2% and 5% for 3 months diminishes concentration of interleukin-10 modulating the inflammatory process and decreased the deposit of collagen attenuated cardiac remodeling after myocardial infarction in rats [54]. Açaí in echocardiographic studies has already been demonstrated to induce functional changes for decreasing systolic fractional area change, ejection fraction, diastolic E’ media and A’ media in myocardial infarction in rats supplemented with 2% or 5% of açaí pulp for 3 months [54] and reduced left ventricular systolic diameter and left atrial diameter with improvement in left ventricular fractional shortening in rats supplemented with açaí 5% for 4 weeks, showing that açaí was effective in improving cardiac function in vivo [31]. In this way, in current study the açaí treatment group showed less toxicity effects and architecture of cardiac tissue maintain. However, it was not possible to analyze the inflammatory markers in the heart, not even the echocardiographic study, which becoming a limitation of the study.

Cardiac toxicity may occur after the administration of some chemotherapy drugs, such as doxorubicin, cyclophosphamide and, 5-fluorouracil [55–57], with a cumulative incidence of 4.1% at 5 years [57], and is the most serious and lethal adverse effect related to chemotherapeutic drugs [55, 57] responsible for 16.3% of deaths in breast cancer patients [57]. Doxorubicin cardiotoxicity, in a previous study, showed that cytoplasm vacuolization was associated with increased interstitial space in histological analysis of rat myocytes [58]. We found that açaí reduced CK, CKMB, troponin I and C-reactive protein levels, decreased H2AX immunostaining and DNA damage markers, and increased collagen and hydroxyproline production, leading to a reduction in heart tissue damage. In addition, there was no cardiac fibrosis in the histopathological analysis of the heart, and the increased collagen production and antifibrosis effect of açaí remain unclear. It is worth mentioning that in our previous study with the same DMBA-induced breast cancer model [21], we observed in the heart histopathological analysis the presence of cardiac muscle tissue preserved with no morphological difference between the saline and açaí groups, demonstrating that açaí is not cardiotoxic. Importantly, açaí treatment in previous studies decreased the cardiotoxicity promoted by doxorubicin treatment in healthy male Swiss albino mice [32] and prevented left ventricular dysfunction and changes in myocardium metabolism in male Wistar rats [31]. Therefore, to our knowledge, this report is the first study to evaluate the combined use of açaí and FAC-D chemotherapy protocols in a breast cancer experimental model. In this way, we propose that açaí administration results in a suppression of chemotherapy-induced toxicity in heart tissues and may be exploited as a promoter of good health, given that it is a safe, low cost and functional food ingredient for combined cancer treatment with chemotherapy.

## Conclusions

In conclusion, açaí displays cardioprotective and anti-inflammatory activities in DMBA-induced breast cancer treated with the FAC-D chemotherapy protocol. Açaí may modulate the cardiotoxicity process of the FAC-D protocol by maintaining normal CK and CK-MB levels, preserving the normal histological structure of heart tissue without causing cardiac DNA damage, and increasing the percentage of collagen fibres in the heart. Furthermore, our results support the use of açaí extract as a complementary treatment for chemotherapy. The mechanism of action of açaí is not completely understood, and further investigations are necessary to elucidate the protective mechanisms of this fruit and to assess its impact on human health.

## Data Availability

All data generated or analyzed during this study are included in this published article.

## Abbreviations

ALT: Alanine aminotransferase
AST: Aspartate aminotransferase
CEUA: Institutional Animal Care and Use Committee
CK: Creatine kinase
CKMB: Creatine kinase MB
DMBA: 7,12-dimethylbenzanthracene
DNA: Deoxyribonucleic acid
H: Histologic scores
HE: Hematoxylin-eosin
UEZO: Universidade Estadual da Zona Oeste
